# Vitamin B_3_ Intercalated in Layered Double Hydroxides: A Drug Delivery System for Metabolic Regulation

**DOI:** 10.1021/acsomega.4c03934

**Published:** 2024-07-17

**Authors:** Caroline Inês Lisevski, Alysson Ferreira Morais, Natasha Fioretto Aguero, Alexandre Candido Teixeira, Francisco Wanderson Moreira Ribeiro, Thiago Carita Correra, Ivan Guide Nunes da Silva, Danilo Mustafa

**Affiliations:** † Instituto de Física da Universidade de São Paulo, 05508-090 São Paulo, SP, Brazil; ‡ Center for Surface Chemistry and Catalysis, KU Leuven, B-3001 Leuven, Belgium; § Department of Fundamental Chemistry, Institute of Chemistry, University of São Paulo, 05508-000 São Paulo, SP, Brazil

## Abstract

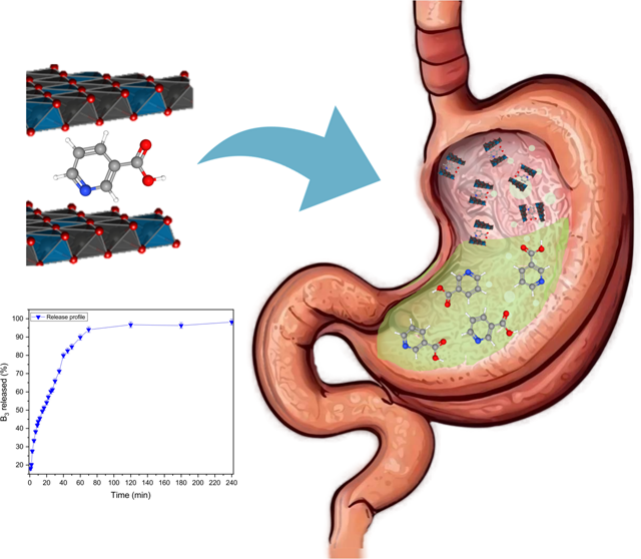

The organic compound niacin or nicotinic acid, also known as vitamin B_3_ (VitB_3_), is essential for human nutrition and metabolic regulation. However, in high doses, it can provoke side effects, such as hyperglycemia, liver damage, and flushing. Development of a controlled release system that slowly releases VitB_3_ into the organism would avoid high dosing peaks, thus contributing to decrease the occurrence of side effects in nutritional supplementation. Here, we show that the slow and controlled release of VitB_3_ in an acid environment can be achieved via its intercalation in layered double hydroxides (LDHs). The synthesis of a ZnAl-VitB_3_ system is shown, in which VitB_3_ is intercalated in a ZnAl LDH. The presence of VitB_3_ in the ZnAl-VitB_3_ system was confirmed by elemental analysis, infrared (FTIR) and NMR spectroscopy, while successful intercalation in the LDHs was revealed by powder X-ray diffraction (PXRD). In vitro release tests were carried out in a concentrated HCl solution of pH 1.5, a pH similar to the human stomach environment. The results showed a steady release of VitB_3_ from the LDH host, with 90% of the vitamin liberated in the first 60 min after the suspension of the LDH in the acidic solution.

## 1 Introduction

**Figure 1:**
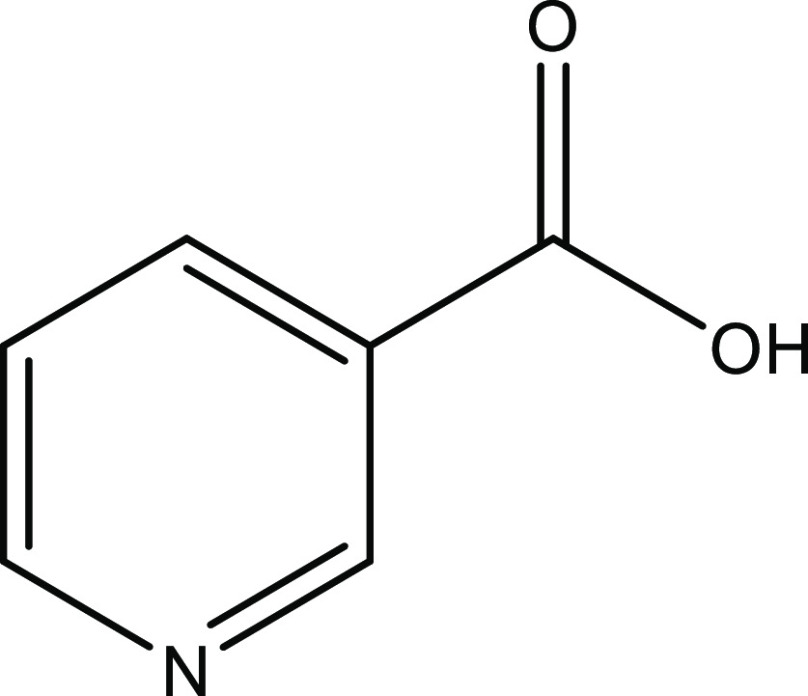
Structural formula of vitamin B_3_ (niacin or pyridine-3-carboxylic acid, p*K*_a_ = 4.85).

Vitamins are organic compounds needed by humans in small amounts for metabolic and physiological functions. As many of them are not synthesized by the human body, they must be obtained from external sources, e.g., from nutrition and/or supplementation. Vitamins can be classified based on their absorption process as fat-soluble vitamins (e.g., Vitamins A, D, E, and K) and water-soluble vitamins (e.g., Vitamin C and all of the B vitamins).^1−3^ Vitamin B_3_, also known as nicotinic acid or niacin, has a molecular formula of C_6_H_5_NO_2_ (Figure 1) and is a water-soluble vitamin, absorbed in the stomach and small intestine. It is naturally found in foods of animal and vegetable origin. Since 1950, it has been used in high doses to treat dyslipidemias (high levels of lipids), and due to its high absorption in the organism, the patient may experience side effects such as hyperglycemia, liver damage, and flushing.^4−6^ These side effects can be avoided if vitamin B_3_ is slowly released into the organism after intake, with the additional benefit of decreasing the necessity of frequent intake by the patient. One way to optimize drug action and minimize side effects is to store the vitamin in a delivery host system that slowly releases it when in contact with the acid solution of the human stomach.^7−9^ In this regard, layered double hydroxides could be used as host systems.

Layered double hydroxides (LDHs) are clay-like layered materials with the general formula [M_1–*x*_^II^ M_x_^III^ (OH)_2_] (A^*n*–^)_*x*/*n*_·*m*H_2_O that can incorporate anionic species (A^*n*–^) in their interlayer space. They are formed by the stacking of M^II^_1–_*_x_*M^III^_*x*_(OH)_2_ double hydroxide layers in which the divalent (M^II^) and trivalent (M^III^) metal cations can be wisely chosen to maintain the biocompatibility of the system. Based on this flexibility in composition, LDHs have been used as delivery systems for anti-inflammatory medication,^10−12^ antibiotics,^13^ vitamins,^14^ and anticancer drugs.^15^

LDHs are easy to prepare via green and biocompatible synthetic routes.^16^ LDHs containing M^II^ = Zn^2+^ or Mg^2+^ and M^III^ = Al^3+^ have already been demonstrated to be biocompatible.^17−20^ LDHs decompose at acid pH, releasing the interlayer anions as the consequence of a controlled dissolution process due to acid attack.^21^ Based on these properties, here we propose the synthesis of a VitB_3_-intercalated LDH composed of Zn^2+^ and Al^3+^ (here, named ZnAl-VitB_3_) and investigate its in vitro controlled release properties in an acidic condition mimicking the pH of the human stomach. The successful uptake of VitB_3_ by the ZnAl-VitB_3_ during synthesis was confirmed by elemental analysis, Fourier transform infrared (FTIR) and NMR spectroscopy, while successful intercalation in the LDH was revealed by powder X-ray diffraction (PXRD) measurements. The VitB_3_ release properties of the system were tested in a concentrated HCl solution of pH 1.5, showing that 90% of the VitB_3_ in the host system is liberated within the first 60 min of contact with the acidic solution.

## 2 Materials and Methods

### 2.1 Materials

All chemicals were purchased and used without further purification, including the metal precursor salts Zn(NO_3_)_2_·6H_2_O (98% mol, Vetec) and Al (NO_3_)_3_·9H_2_O (98% mol, LabSynth), nicotinic acid (VitB_3_, 97% mol, Vetec), and chloridric acid (HCl, 37% vol, Sigma-Aldrich).

### 2.2 Synthesis

Intercalation of VitB_3_ in the LDHs was achieved by coprecipitation of Zn^2+^ and Al^3+^ at constant pH in a solution containing dissolved VitB_3_.^22^ A 0.7 mol·L^–1^ solution of VitB_3_ was prepared by dissolving VitB_3_ in 200 mL of deionized water and adjusting the pH to pH 8 to deprotonate its acid group. The LDHs intercalated with VitB_3_ were prepared by dosing into the VitB_3_ solution 10 mL of a metal solution containing 0.333 mol·L^–1^ of Al(NO_3_)_3_·9H_2_O and 0.666 mol·L^–1^ of Zn(NO_3_)_2_·6H_2_O. During dosing, the pH of the VitB_3_ solution was stated to pH 8 by an automatic titrator (Titrino 702 SM, Metrohm, Switzerland) that controlled the dosing of a 1 mol·L^–1^ NaOH solution in the synthesis pot. The solution was stirred at a constant stirring of around 100 rpm during the synthesis. After the dosing of the metals was finished, the produced slurry was stored in an oven at 60 °C for 2 days to optimize crystallization. After that, the solid phase was recovered by centrifugation and washed with deionized water several times to dilutions of more than 100 times to remove residual ions. The solid was subsequently dried at 60 °C for 4 days in an oven.

### 2.3 Characterization

XRD measurements were performed in the Bragg–Brentano geometry on a D8 Discover diffractometer (Bruker) equipped with a Cu Kα radiation source (λ = 1.5418 Å, 40 kV and 30 mA) and a Lynxeye detector. The 2θ angle between the incidence and the detection directions ranged from 4 to 70° in steps of 0.05° using an integration time of 1.5 s while the sample was rotated perpendicularly to the incident beam at a rate of 20 rpm. CHN elemental analysis was performed in a PerkinElmer 2400 series ii Elementary Analyzer to determine the concentration of carbon, hydrogen, and nitrogen (CHN) through the Pregl-Dumas method. Inductively coupled plasma optical emission spectrometry (ICP-OES) was performed in a Spectro Arcos analyzer (SPECTRO Analytical Instruments GmbH, Germany) to analyze the Zn and Al contents of the LDH sample. Thermogravimetric analysis was carried out using a Thermogravimetric analysis (TGA) Q500 (TA Instruments) from room temperature up to 800 °C at a heating rate of 5 °C min^–1^ using an airflow of 60 mL min^–1^. The Fourier transform infrared (FTIR) spectrum shown was recorded in the range 400 to 4000 cm^–1^ in a PerkinElmer Frontier FTIR spectrometer using sample-embedded KBr pellets. Nuclear magnetic resonance spectroscopy was performed in a Bruker Avance II+ 400 MHz spectrometer equipped with a 4 mm triple resonance solid-state magic angle spinning (MAS) probe. ^1^H direct excitation and ^1^H–^13^C CPMAS spectra were acquired at 22 °C at a MAS rate of 15 kHz. For the measurement, the sample was packed in a 4 mm ZrO_2_ rotor. Mass spectrometry (MS) experiments were performed using a linear quadrupole ion trap MS (LTQ XL, Thermo Scientific, San Jose, CA) equipped with a heated electrospray source (HESI) using N_2_ as nebulizer, sheath, and dry gas (Peak Scientific, NM32LA model). The mass spectrometry data was obtained by direct infusion of the samples solubilized and diluted in acetonitrile at a final concentration of vitamin B_3_ of 10^–5^ mol L^–1^ at a flow rate of 3 μL min^–1^. The solutions containing LDH were filtered (0.45 μm PTFE filter) before analysis. The MS spectra were acquired in positive mode with a mass scan range of *m*/*z* 60–140, a maximum inject time of 10 ms, and averaging 5 spectra. The spray voltage was 4.0 kV, the temperature was set at 275 °C, and the dry gas (N_2_) flow rate was 15 arb.

### 2.4 In Vitro Drug Release

The drug release profile was determined in an in vitro assay. The experiment was performed using an HCl aqueous solution of pH 1.5 at 37 °C and with magnetic stirring at 100 rpm. 200 mg of ZnAl-VitB_3_ were dissolved in 200 mL of this HCl solution, and, at different time intervals, 2 mL aliquots were removed, and the same volume of the original HCl solution was replenished. This procedure was repeated until the maximum time of 240 min was reached. The concentration of vitamin B_3_ in the aliquots was determined by ultraviolet–visible (UV–vis) spectrophotometry using a double beam UV-2700i (Shimadzu) equipped with a double monochromator previously calibrated for VitB_3_ determination using standard VitB_3_ solutions of known concentrations. The spectra were analyzed in the range of 260 to 400 nm.

## 3 Results and Discussion

The intercalation of VitB_3_ within the hydroxide layers of LDHs was achieved here (sample dubbed ZnAl-VitB_3_) by a coprecipitation procedure in which nitrate salt precursors of Zn^2+^ and Al^3+^ were dropwise added to a solution containing VitB_3_ deprotonated by stating the pH of this solution to pH 8. To achieve high VitB_3_ loading in the solid, a high Al/(Al+Zn) ratio of 0.33 was used in the composition of the solids, this ratio representing the maximum M^III^ loading that can be obtained using ambient conditions. More details on the experimental procedure are available in Section 2.

**Figure 2:**
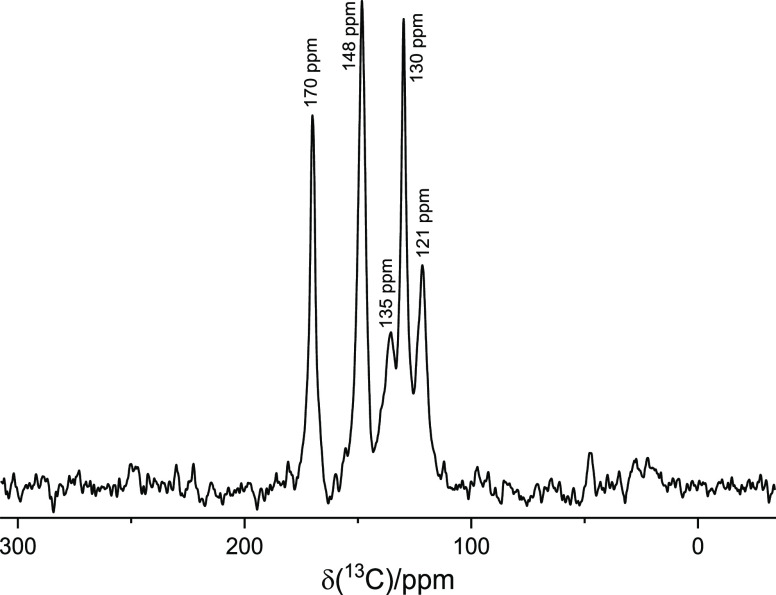
^1^H decoupled ^1^H–^13^C CPMAS NMR spectrum of the ZnAl-VitB_3_ solid acquired at 22 °C under an MAS rate of 15 kHz. Magnetic field: 9.4 T (^1^H frequency: 400 MHz).

**Figure 3:**
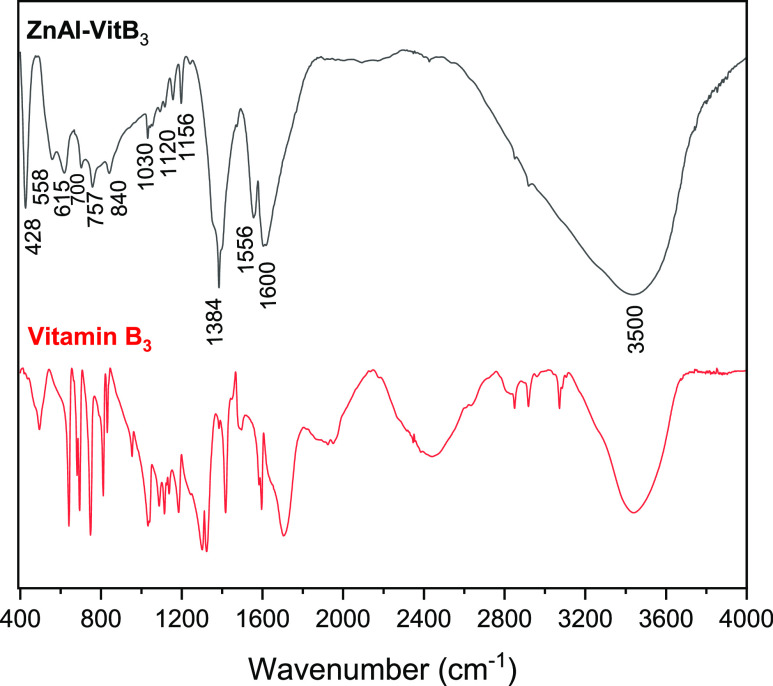
FTIR spectrum of as-purchased Vitamin B_3_ and ZnAl-VitB_3_. The numbers represent the center (in cm^–1^) of the major absorption bands observed in the spectrum of ZnAl-VitB_3_.

The presence of VitB_3_ in the ZnAl-VitB_3_ solid after washing to remove unbound and unreacted salts was examined via ^1^H–^13^C CPMAS NMR (Figure 2) and FTIR (Figure 3). The ^1^H–^13^C CPMAS NMR spectrum confirms the presence of VitB_3_ in the sample, with the presence of the typical ^13^C NMR resonances of VitB_3_ appearing at chemical shifts δ_C_(400 MHz) 121, 130, 135, 148, and 170 ppm (lit., 123.71, 126.66, 136.90, 150.21, and 166.23 ppm^23^). The resonance observed at 170 ppm can be attributed to the carbonyl (C=O) group, whereas the remaining resonances are associated with carbon atoms within the aromatic ring structure.

The FTIR spectrum of ZnAl-VitB_3_ (Figure 3) exhibits prominent peaks and well-defined absorption bands corresponding to the functional groups present in the LDH solid, also confirming the presence of VitB_3_ in the sample. The broad band observed at approximately 3500 cm^–1^ corresponds to the stretching mode of the hydroxyl (O–H) groups and water (H_2_O) molecules. The peak centered around 1600 cm^–1^ corresponds to the stretching mode of C=C bonds of VitB_3_, while the peak at 1556 cm^–1^ arises from N–O stretching bonds of the nitrate anions co-intercalated in the LDH. The peak at 1384 cm^–1^ corresponds to the antisymmetric stretching mode (ν_3_) of the nitrate anion, and the peak at 1197 cm^–1^ is associated with C–O stretching of carboxylate from VitB_3_. The band ranging from 1156 to 1030 cm^–1^ is attributed to in-plane C–H vibrations, while the band spanning from 700 to 615 cm^–1^ is ascribed to out-of-plane C–H vibrations, both ascribed to the pyridine ring of VitB_3_. Furthermore, the peaks observed at 840 cm^–1^, 750 cm^–1^, and 558 cm^–1^ can be attributed to the weak out-of-plane symmetric deformation mode (ν_2_) of nitrate, the Al–OH deformation, and the Zn/Al–OH translation, respectively.^24−^

The elemental composition of ZnAl-VitB_3_ was examined by using ICP-OES and CHN analysis. The empirical formula of the precipitate was derived based on specific assumptions: first, that carbon solely originates from vitamin B_3_; and second, that nitrogen is sourced from nitrate ions within the samples. The resulting empirical formula is: **ZnAl-VitB**_**3**_. Anal. Calcd for [Zn_2_Al_0.95_(OH)_5.9_][(NO_3_^–^)_0.24_(VitB_3_^–^)_0.71_]·2.0 H_2_O wt % Calc.: Zn, 33.17; Al, 6.50; C, 12.96; H, 3.23; N, 3.37. wt % Found: Zn, 34.23; Al, 6.71; C, 12.8; H, 3.3; N, 3.1. Based on this data, the total amount of VitB3 on LDHs corresponds to 22.2 wt %.

As suggested by the experimental formula, most of the layer charge of the LDHs is compensated by intercalation of VitB_3_ in the LDHs, while nitrate is still present in a considerable amount. The M^II^/M^III^ metal fraction for ZnAl-VitB_3_ (2.1) is in accordance with the nominal value (2.0) expected from the Zn and Al stoichiometries initially added during synthesis.

**Figure 4:**
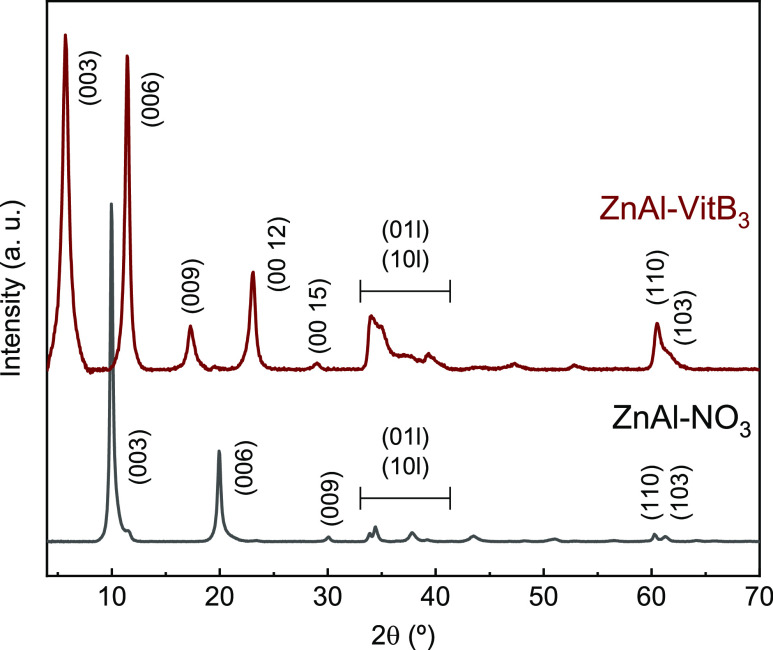
PXRD patterns for a nitrate-intercalated (ZnAl-NO_3_) and Vitamin B_3_-intercalated ZnAl-VitB_3_ LDH sample. Data were measured using Cu Kα radiation with λ = 1.5418 Å. The diffraction patterns have been indexed based on a 3-layer hexagonal unit cell.

The crystalline structure of ZnAl-VitB_3_ was examined using PXRD, as depicted in Figure 4. A comparison was made between the PXRD diffractogram of ZnAl-VitB_3_ and a purely nitrate-intercalated Zn^2+^/Al^3+^ LDH sample investigated by us in a previous study (here, dubbed ZnAl-NO_3_).^28^ For LDHs, the interlayer distance calculated from the main Bragg reflection at low 2θ can be used to assess the successful interaction of the desired anion. Nitrate-intercalated LDHs exhibit an intense Bragg reflection at 9.97° 2θ, corresponding to the (003) crystalline plane, with a basal spacing of 8.905 Å, characteristic of nitrate-intercalated LDH materials.^29^ In the case of ZnAl-VitB_3_, the Bragg reflection corresponding to this same crystalline plane appears at 5.70° 2θ, accompanied by an increased basal spacing of 15.5 Å, along with broadening of the peak. These observations indicate the intercalation of vitamin B_3_ within the LDH structure.^29^ Accounting for the thickness of a hydroxide layer (∼4.8 Å^30^), the interlayer distance matches with the longitudinal dimensions of VitB_3_. This implies a perpendicular positioning of this interlayer anion relative to the hydroxide layers of the LDHs. From the (110) reflection at 60.45° 2θ, the metal–metal distance within the hydroxide layers can be calculated: “*a* = 2d_(110)_ = 3.06 Å”.

**Figure 5:**
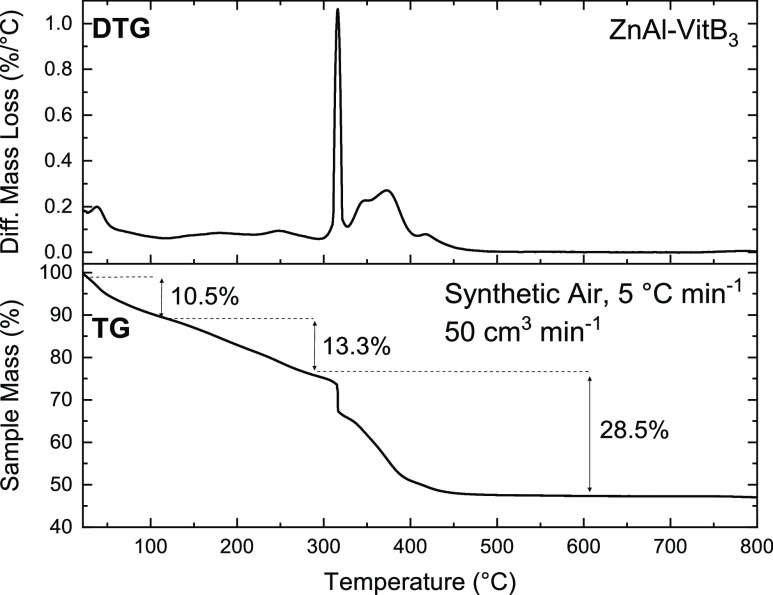
Thermogravimetric analysis of ZnAl-VitB_3_ showing the thermal stability of the material and its decomposition events at temperatures of up to 800 °C.

The thermal stability of the new ZnAl-VitB_3_ solid was investigated using thermogravimetry, and the intensity of the observed mass loss events in the TGA curve was compared against the proposed chemical formula derived from elemental analysis. The TGA curve (Figure 5) shows a 10.5% decrease in the weight of the sample when the temperature is ramped up to 120 °C, which is attributed to the removal of superficial and coordination water intercalated in the interlayers of the LDHs (Calc. 9.1%). The continued mass loss from 120 to 290 °C is mainly attributed to the dehydroxilation of the hydroxide layers, with the formation of mixed oxyhydroxides and the release of water vapor (Exp. 13.3%, Calc. 13.4%).^31^ The decomposition process of vitamin B_3_ and nitrate intercalated in the LDHs mainly occurs between 290 and 490 °C, observed in the derivative thermogravimetric (DTG) curve as two intense mass loss events (Exp. 28.5%, Calc. 25.8%).^32,33^

**Figure 6:**
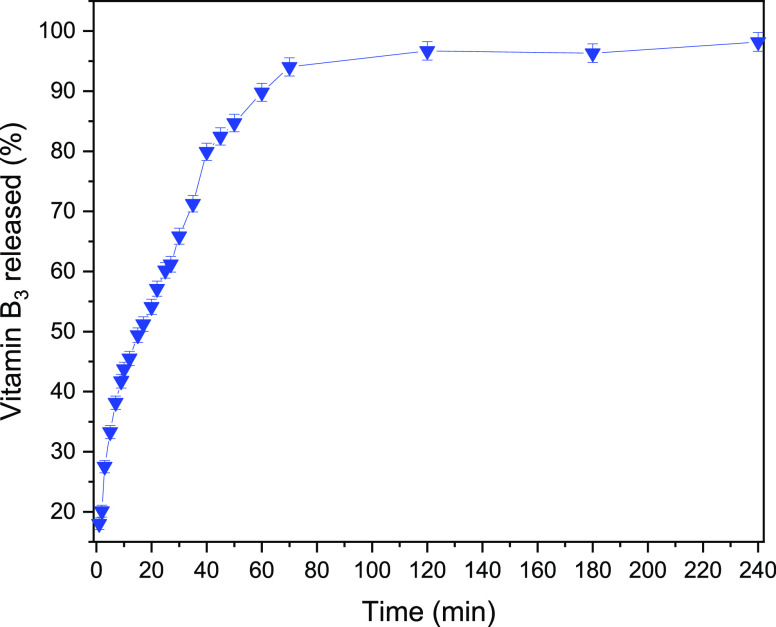
Drug release profile measured as the amount of Vitamin B_3_ released from the solid ZnAl-VitB_3_ phase when this sample is suspended and stirred in an aqueous HCl solution of pH = 1.5.

To investigate the in vitro release of vitamin B_3_ in a condition mimicking the pH of the human stomach, the ZnAl-VitB_3_ solid was dispersed in an HCl solution of pH 1.5 under continuous stirring. The decomposition of the LDH host under acidic conditions leads to the release of interlayer contents. During the experiment, aliquots of 2 mL were collected at various time intervals until a maximum duration of 240 min. Following calibration, the release profile (Figure 6) was determined by measuring the absorbance of vitamin B_3_ (see also Figure S1). The release kinetics demonstrate an initially rapid process, with 50% of the Vitamin B3 loaded in the LDH being released at *T*_50_ ∼ 20 min. Subsequently, the release rate gradually decreases, with 75 and 90% of the intercalated vitamin B_3_ being released after *T*_75_ ∼ 40 min and *T*_90_ ∼ 65 min, respectively.

**Figure 7:**
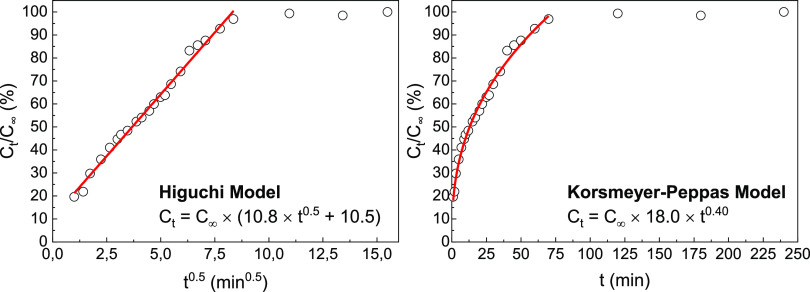
Fittings of the drug release profile using (left) the Korsmeyer-Peppas model and (right) the Higuchi model.

Finding a suitable mathematical model to describe a set of experimental drug release data is crucial in pharmaceutical research. Such a model not only grants an understanding of drug behavior upon release from diverse delivery systems but also enables predictions. By effectively characterizing drug release kinetics, drug formulations, dosage regimens, and delivery mechanisms can be optimized, ultimately leading to enhanced therapeutic outcomes. Among the different models available, the zero-order and first-order fits (see also Figure S2), and the widely used Higuchi and Korsmeyer–Peppas models were considered.^34^ For the release until 70 min, the Higuchi and Korsmeyer–Peppas models emerge as the best fits for the release data of VitB_3_ in the LDH system (Figure 7). The Higuchi model can be expressed by the equation
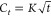
1where *C_t_* represents the drug concentration at a given time *t* and *K* is the release constant. The release constant *K*, influenced by factors such as the drug’s diffusivity, matrix membrane thickness, and others, provides insights into the underlying diffusion-driven drug release process. Higher *K* values indicate a faster drug release; lower *K* values suggest a slower drug release. For our specific case, the fitted value of *K* for the Higuchi model is 10.8 ± 0.2 min^–0.5^, in the range usually referred to as a slow-release profile.^34^

The Korsmeyer–Peppas model is described by
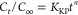
2where *C_t_* represents the drug concentration at a given time *t*, *C*_∞_ represents the total amount of the drug in the equilibrium, *K*_KP_ is the Korsmeyer–Peppas constant, and *n* is the release exponent, which is used to characterize the different release mechanisms. If *n* ≤ 0.45, the release mechanism is predominantly Fickian diffusion; for 0.45 < *n* < 0.89, it is non-Fickian diffusion; *n* = 0.89 is a Case II transport and *n* > 0.89 a super Case II transport.^35^ Fickian diffusion refers to the molecular process where the mass transfer rate is proportional to the concentration gradient (Fick’s laws). This type of diffusion is characteristic of systems in which particle movement occurs in a relatively slow and predictable manner, typical in solids and low-concentration liquids. Case II transport, otherwise, does not follow Fick’s law and usually occurs on polymers where the diffusion is also dependent on the polymer relaxation dynamics. By fitting the data with the Korsmeyer–Peppas model, *n* = 0.399 ± 0.010 is obtained, which indicates a predominantly Fickian diffusion.^36^

To further verify the integrity of Vitamin B_3_ under the conditions applied during the synthesis of LDH and during the release experiment, mass spectrometry was employed to analyze the presence of Vitamin B_3_ in the solution obtained after the release experiment. Figure S3a shows the mass spectrum of as-purchased Vitamin B_3_ in water before any kind of treatment. The protonated species [VitB_3_ + H]^+^ appears at *m*/*z* 124. Figure S3b shows the mass spectrum of the solution containing Vitamin B_3_-intercalated LDH after filtering the solution with a 0.45 μm PTFE filter. No relevant signals were observed in the same intensity range as that in Figure S3a, thus showing the immobilization of Vitamin B_3_ in the LDH phase. Figure S3c shows the mass spectrum of the solution resulting from the acid treatment of the ZnAl-VitB_3_ LDH sample. Vitamin B_3_ is again observed in a relative intensity similar to what was observed in Figure S3a. This shows not only that Vitamin B_3_ is still present in solution after acid treatment but also that it has not degraded.

## 4 Conclusions

In summary, the controlled release of vitamin B_3_ in in vitro conditions mimicking the human stomach can be achieved by intercalating vitamin B_3_ in layered double hydroxides. Intercalation can be achieved by coprecipitation of the drug in the presence of Zn^2+^ and Al^3+^. When dispersed in concentrated HCl solution with pH 1.5, the LDH host is decomposed and 90% of the vitamin is liberated to the solution within a timelapse of 60 min. These findings turn ZnAl-VitB_3_ into a potential VitB_3_ delivery system. Further investigations will explore its performance in different physiological environments and its potential applications in pharmaceutical formulations, as well as explore other anion combinations to harness the potentially harmful effect of nitrate in the intestine.

## Supporting Information Available

Calibration curve for the drug release test; zero-order and first-order model fittings for the release profile; and mass spectrometry for the VitB_3_ and ZnAl-VitB_3_ before and after the acid treatment (PDF)
