# Supplementary material for: Vitamin B3 Intercalated in Layered Double Hydroxides: A Drug Delivery System for Metabolic Regulation
Source: ACS Omega. 2024 Jul 17;9(30):32962–8. doi: 10.1021/acsomega.4c03934 (PMC11292643; doi:10.1021/acsomega.4c03934)
Supplement: Supplementary file 1 — ao4c03934_si_001.pdf [file ao4c03934_si_001.pdf]

Supporting Information

Vitamin B_3 intercalated in layered double hydroxides: a drug delivery system for metabolic regulation

Caroline Inês Lisevski[1], Alysson Ferreira Morais[1,2], Natasha Fioretto Aguero[1], Alexandre Candido Teixeira[1], Francisco Wanderson Moreira Ribeiro[3], Thiago Carita Correra[3], Ivan Guide Nunes da Silva[1] and Danilo Mustafa[1,*]

[1] Instituto de Física da Universidade de São Paulo, 05508-090 - São Paulo, SP, Brazil
[2] Center for Surface Chemistry and Catalysis, KU Leuven, B-3001, Leuven, Belgium
[3] Department of Fundamental Chemistry, Institute of Chemistry, University of São Paulo, 05508-000 - São Paulo, SP, Brazil.

* Corresponding Author: dmustafa@if.usp.br

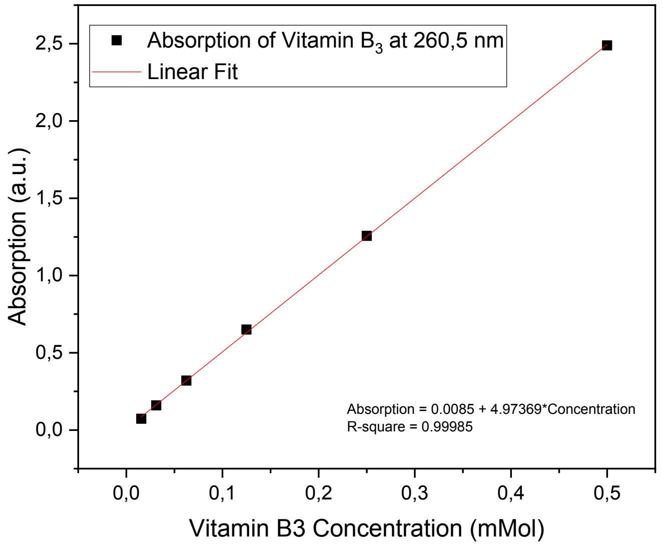

Figure S1. Calibration curve for quantification of Vitamin B_3 using absorbance in buffer HCl. The absorption peak occurs at 260.5 nm.

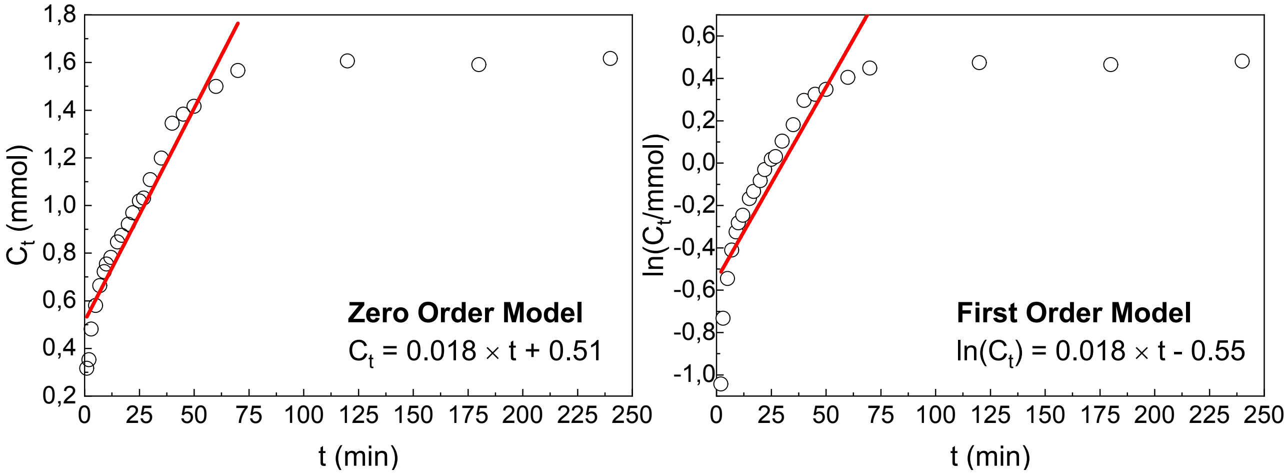

Figure S2. Release models fitting for vitamin B_3 release profile: (left) Zero Order Model and (right) First Order Model.

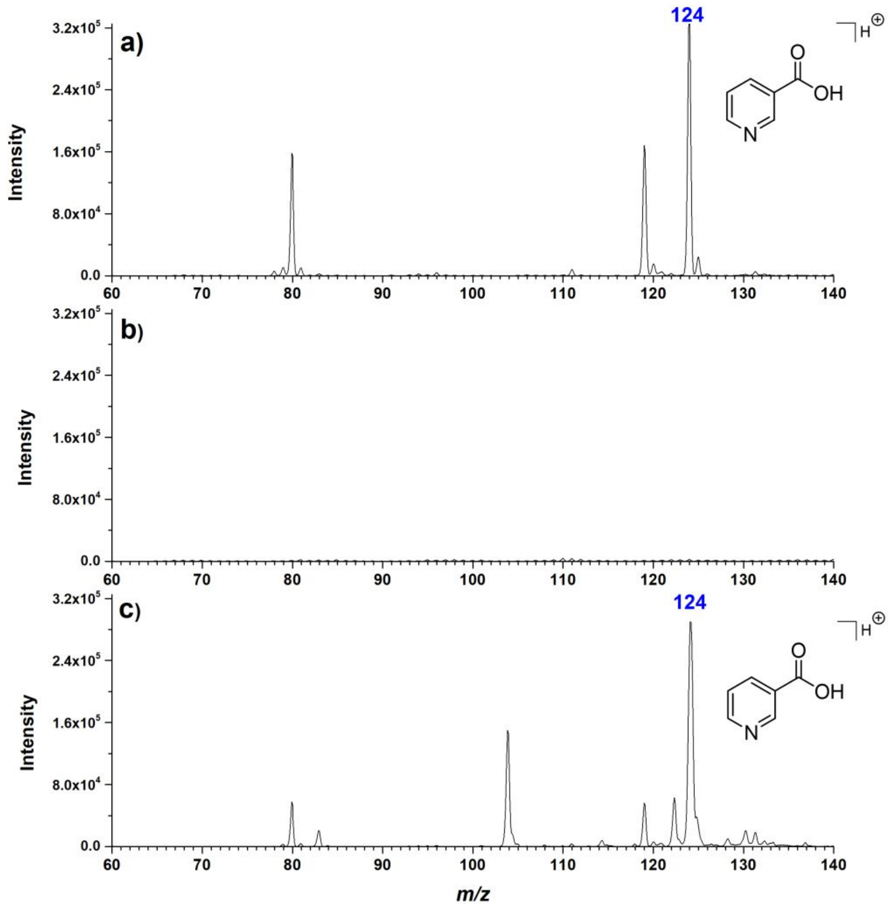

Figure S3. Mass spectrum for a) as purchased Vitamin B_3 (the signal at *m/z* 119 corresponds to $[VitB3]^+$), b) An aqueous dispersion of the ZnAl-VitB_3 sample after filtering (0.45 μm PTFE filter) to remove the solid phase, and c) the liquid phase resulting after acid treatment of the ZnAl-VitB_3 sample in a solution of pH 1.5 (see details in **Materials and Methods**).
